# Acro-cardio-facial syndrome

**DOI:** 10.1186/1750-1172-5-25

**Published:** 2010-09-29

**Authors:** Maria Cristina Digilio, Bruno Dallapiccola

**Affiliations:** 1 Division of Medical Genetics, Bambino Gesù Pediatric Hospital, IRCCS, Piazza S. Onofrio 4, 00165, Rome, Italy

## Abstract

Acro-cardio-facial syndrome (ACFS) is a rare genetic disorder characterized by split-hand/split-foot malformation (SHFM), facial anomalies, cleft lip/palate, congenital heart defect (CHD), genital anomalies, and mental retardation. Up to now, 9 patients have been described, and most of the reported cases were not surviving the first days or months of age. The spectrum of defects occurring in ACFS is wide, and both interindividual variability and clinical differences among sibs have been reported. The diagnosis is based on clinical criteria, since the genetic mechanism underlying ACFS is still unknown. The differential diagnosis includes other disorders with ectrodactyly, and clefting conditions associated with genital anomalies and heart defects. An autosomal recessive pattern of inheritance has been suggested, based on parental consanguinity and disease's recurrence in sibs in some families. The more appropriate recurrence risk of transmitting the disease for the parents of an affected child seems to be up to one in four. Management of affected patients includes treatment of cardiac, respiratory, and feeding problems by neonatal pediatricians and other specialists. Prognosis of ACFS is poor.

## Disease name and synonyms

Acro-cardio-facial syndrome (ACFS)

Cleft palate-cardiac defect-genital anomalies-ectrodactyly (CCGE) syndrome

## Definition

Acro-cardio-facial syndrome (ACFS, OMIM 600460) is a rare genetic disorder characterized by split-hand/split-foot malformation (SHFM), facial anomalies, cleft lip/palate, congenital heart defect (CHD), genital anomalies, and mental retardation. This association was first described by Richieri-Costa and Orquizas in 1987 in a Brazilian patient born to consanguineous parents [1]. The existence of this syndrome was corroborated by the report of 8 additional patients [2-7]. We are aware of an additional Turkish patient born to consanguineous parents (Kayresili, personal communication 2009), presenting with SHFM, aortic stenosis, cleft palate, hypoplastic corpus callosum, and atretic ears. The acronym CCGE (cleft palate-cardiac defect-genital anomalies-ectrodactyly) was also proposed to identify this unique constellation of anomalies [2]. Additional features occasionally found in these individuals include cortical atrophy of the brain [3], cerebral neuroepithelial cyst [5], growth retardation [1-3,6], vertebral malformations [7], subclinical hyperthyroidism [7], imperforate anus [6]. ACFS is a lethal disorder as shown by the early demise in the first months of life in most of these patients.

## Epidemiology

The incidence of ACFS has not been determined, due to the paucity of the reported cases, but it is likely a very rare disease (<< 1 in 100,000 newborns). A similar occurrence among genders is expected for an autosomal disorder. The excess of male patients reported so far (7 M: 3 F) is likely biased by the low number of observations.

## Clinical description

Clinical features of ACFS are summarized in Table 1. The most important diagnostic handles are SHFM and CHD. The spectrum of defects occurring in ACFS is wide, and both interindividual variability and clinical differences among sibs have been documented. Most published cases have not survived the first days or months of life.

**Table 1:** Major clinical features of the acro-cardio-facial syndrome

Clinical finding	References									Total %	
	1	2a	2b	3	4	5a	5b	6	7		
**Sex**	**M**	**M**	**F**	**M**	**M**	**M**	**F**	**F**	**M**	**6 M/3F**	
											
Facial anomalies	+	+	+	+	+	+	+	+	+	9/9	100
High forehead	-	+	NK	-	+	+	+	NK	+	5/7	71
Prominent eyes	-	-	NK	+	+	-	-	NK	-	2/7	29
Long eyelashes	+	-	NK	+	+	-	-	NK	-	3/7	43
Hypertelorism	-	-	NK	+	+	+	+	NK	+	5/7	71
Broad/flat nasal root	+	+	NK	+	+	-	-	NK	+	5/7	71
Cleft lip	+	-	-	+	-	-	-	-	+	3/9	33
Cleft palate	+	-	+	+	-	-	NK	+	+	5/8	63
Low-set dysmorphic ears	+	+	NK	+	+	+	+	+	+	8/8	100
											
Limb anomalies	+	+	+	+	+	+	+	+	+	9/9	100
Cleft hand (bilateral)	+	+	+	+	-	+	+	+	-	7/9	78
Cleft hand (unilateral)	-	-	-	-	+	-	-	-	+	2/9	22
Cleft foot (bilateral)	+	-	-	-	-	-	+	-	-	2/9	22
Cleft foot (unilateral)	-	-	-	-	+	-	-	-	-	1/9	11
											
Congenital heart defect	+	+	-	+	+	-	-	+	+	6/9	67
Ventricular septal defect	+	+	-	-	-	-	-	+	-	3/9	33
Atrial septal defect	-	+	-	-	-	-	-	+	-	2/9	22
Aortic coarctation	-	+	-	+	-	-	-	-	-	2/9	22
Mitral atresia	-	+	-	-	-	-	-	-	-	1/9	11
Truncus arteriosus	-	-	-	-	+	-	-	-	-	1/9	11
Tetralogy of Fallot	-	-	-	-	-	-	-	-	+	1/9	11
Absent left pulmonary artery	-	-	-	-	-	-	-	-	+	1/9	11
											
Genital anomalies	+	+	NA	-	+	+	NA	NA	+	5/6	83
Micropenis	+	+	NA	-	-	+	NA	NA	+	4/6	67
Cryptorchidism	+	+	NA	-	+	+	NA	NA	-	4/6	67
Hypospadia	+	+	NA	-	-	+	NA	NA	-	3/6	50
											
Low birth weight (< 3rd centile)	-	+	+	-	-	+	+	-	+	5/9	56
Growth retardation	+	+	NA	+	+	-	NA	NK	+	5/6	83
Neurological anomalies	+	+	NK	+	+	-	NK	+	+	6/7	86
Mental retardation	+	+	NK	+	+	-	NK	NK	+	5/6	83
Hypotonia/Hypertonia	+	+	NK	-	+	-	NK	NK	-	3/6	50
Cortical brain atrophy	+	-	-	+	NK	NK	NK	-	NK	2/5	40
Neuropepithelial cyst	-	-	-	-	NK	NK	NK	+	NK	1/5	20
											
Dead/Alive	Alive	Dead	Dead	Dead	Dead	Alive	Dead	Dead	Dead		
Age	4 y	1 m	6 h	4 m	1 m	25 y	4d	50 d	14 y		

References: 1, Richieri-Costa and Orquizas, 1987 [1]; 2, Giannotti et al., 1995 [2]; 3, Guion-Almeida et al., 2000 [3]; 4, Mingarelli et al., 2005 [4]; 5, Sivasli et al., 2007 [5]; 6, Kariminejad et al., 2008 [6]; 7, Tanpaiboon et al., 2009 [7]

Abbreviations: M, Male; F, female; NK, not known; NA, not applicable; y, years; m, months; h, hours; d, days

## Limbs

**Figure 1:**
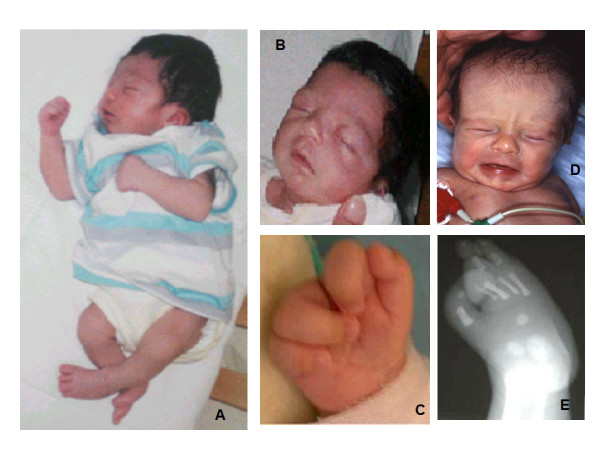
**Facial and limb features of two patients with the acro-cardio-facial syndrome: A, B, C, E: patient reported by Mingarelli et al., 2005 **[4]**; D: patient reported by Giannotti et al., 1995 **[2]**(2a in Table 1)**. A: Lateral total body view; B: Facial appearance of patient 4, showing high forehead, prominent eyes, long eyelashes, hypertelorism, broad nasal root; C: Ectrodactyly of right hand; D: Facial appearance of patient 2a, showing high forehead, broad nasal root, low-set dysmorphic ears; E: X-ray of right foot, showing split foot malformation with five metatarsal (including a very hypoplastic second metatarsal) and absence of all the phalanges of second and third toes.

Cleft hand is a constant feature, either bilateral or unilateral (figure 1C). Subluxation of metacarpophalangeal joints and finger flexion at the proximal interphalangeal joints can be also found [6]. Cleft foot occurs in a proportion of these patients. Cutaneous finger and toe syndactyly has been reported in some cases [3-5].

Radiographic findings include cleft hand/foot with agenesis of fingers [1-7] (figure 1E), abnormal structure and articulation of the first metacarpal bone [4,6,7], hypoplastic and short metacarpal bones [1-7], presence of extrabones between phalanges [6,7], hallux valgus [4], absent or abnormally modelled phalanges of toes [4], polydactyly of foot [5].

## Congenital heart defects

CHD have been detected in two third of the patients [1-5,7]. Anatomic types are heterogeneous, including septal defects [1,5], left-sided obstructive lesions [2,3], and conotruncal defects [4,7]. Left-sided obstructive lesions have been described, as aortic coarctation and hypoplastic left heart. Conotruncal heart defects include truncus arteriosus type 1 with dysplastic and stenotic truncal valves [4] and tetralogy of Fallot with absent left pulmonary artery [7].

## Facial anomalies

Facial anomalies are not distinct for the syndrome, and clinical expression appears quite variable. Reported dysmorphisms include high forehead, prominent eyes, long eyelashes, hypertelorism, flat nasal root, low-set dysmorphic ears (figure 1A, B, D). Lip and palate anomalies are often present, manifesting as bilateral cleft lip and cleft palate [1,7], unilateral cleft lip and palate [3], cleft palate only [2,5]

## Genital anomalies

Male patients manifest different genital anomalies, ranging from micropenis [1,2,6,7] to cryptorchidism [1,2,4,6], and hypospadia [1,2,6].

## Growth

Growth retardation is a common prenatal and postnatal finding, resulting in low birth weight [2,6,7], weight deficiency often in conjunction with feeding difficulties [1-3,7], and short stature [1,6,7].

## Neurological anomalies

The prevalence of mental retardation in ACFS is at present unknown, due to early demise of the majority of these patients. One of the two survivors was mentally normal by age 25 years [6], while the other displayed developmental delay at the age of 4 years [1]. A patient described by Tanpaiboon et al [2009] [7], who died at age of 14 years, was mentally normal. Neurological anomalies as hypotonia, hypertonia, and seizures have been reported in the first days/months of life. Occasional brain anomalies have been observed, including cortical atrophy [1,3] and cerebral neuroepithelial cyst [5].

## Etiology

The genetic mechanism underlying ACFS is still unknown. Isolated or syndromic SHFM has been linked to different loci or genes. Mutations in *p63 *gene, responsible for Ectrodactyly-Ectodermal defects-Cleft (EEC) syndrome and related disorders with SHFM [8], have been excluded in a patient with ACFS [7]. An autosomal recessive pattern of inheritance is supported both by consanguinity in a few families and recurrence in sibs born to unaffected parents [1,2,6]. The involvement of a small chromosomal microdeletion cannot be ruled out, since CGHarray has never been performed in published patients. The possibility should also be considered that this condition could be genetically heterogeneous.

## Diagnosis

The diagnosis of ACFS is solely based on clinical characteristics. The major diagnostic criteria include SHFM and CHD. Cleft lip/palate and genital anomalies are less common features. Although facial anomalies are not distinct, low-set dysmorphic ears appear as a constant feature. Recurrence among sibs born to unaffected parents suggests an autosomal recessive inheritance and provides a clue to differentiate ACFS from autosomal dominant disorders with similar features.

## Antenatal diagnosis

The major diagnostic features characteristic of ACFS can be detected prenatally by ultrasonography. A second trimester scan, including echocardiography and upper/lower limbs evaluation is recommended for monitoring the pregnancies of parents with an affected child.

## Genetic counselling

An autosomal recessive pattern of inheritance has been established for ACFS. Therefore, the more appropriate recurrence risk of transmitting the disease for the parents of an affected child seems to be up to one in four.

## Differential diagnosis

ACFS shares features with other ectrodactyly syndromes and clefting conditions associated with genital anomalies. However, EEC syndrome [9], Rapp-Hodgkin syndrome [10] and ectrodactyly-cleft lip/palate-hand/foot deformities-mental retardation [11,12] can be ruled out, based on lack of ectodermal involvement in ACFS. Malpuech syndrome can be also excluded based on distinct facial features and absent limb defects [13]. CHD, cleft palate, and genital anomalies are features of genito-palato-cardiac syndrome, but none of the reported cases had ectrodactyly [14]. DiGeorge/velo-cardio-facial syndrome can be considered in patients with conotruncal heart defect [4,7], and excluded using FISH analysis of chromosome 22q11.2 [4]. CHARGE syndrome can also be included among conditions in differential diagnosis, since SHFS can be found and ears can be similar in the two conditions [3].

## Management

Patients with ACFS are at high risk of death in the first months of age. The co-occurrence of CHD, low birth weight and hypotonia probably play a major role onto the weakness of affected patients. Cardiac and respiratory problems should be treated by the neonatologist and other specialists, including the cardiologist and the broncopneumologist. A nutrition specialist should be consulted for feeding problems. The surviving patients will benefit from physical therapy, which should start in the first months of life in babies manifesting hypotonia/hypertonia and motor delays. In the surviving patients, neuropsychological assessment should be performed every year to check for the presence of developmental and cognitive delay. Deficits should be treated with rehabilitative programs. The correction of SHFM depends on the individual anatomic defects, and should be managed by orthopaedics and plastic surgeons. When indicated, genital anomalies should be treated by surgeons or urologists.

## Prognosis

Life expectancy is very poor in ACFS individuals. Most of the known patients survived only a few hours or months. Cardiopulmonary complications were the main cause of death. Two patients were alive at time of the observation, respectively at 4 and 25 years.

## Abbreviations

ACFS: acro-cardio-facial syndrome; CCGE: cleft palate-cardiac defect-genital anomalies-ectrodactyly; CHD: congenital heart defect; EEC: ectrodactly-ectodermal defect-clefting; OMIM: Online Mendelian Inheritance in Man; SHFM: split-hand/split-foot malformation.

## Consent

Written informed consent was obtained from the patients' parents for publication of these case reports and accompanying images. A copy of the written consent is available for review by the Editor-in Chief of this journal.

## Competing interests

The authors declare that they have no competing interests.

## Authors' contributions

MCD and BD have revised the literature and drafted the manuscript. All authors have read and approved the final manuscript.
